# Uncovering the potential of biofabricated *Ananas comosus* peel selenium nanoparticles for antibacterial, antibiofilm, suppression of virulence genes (can and LuxS), anticancer, and antioxidant properties

**DOI:** 10.1186/s12896-025-00999-x

**Published:** 2025-06-28

**Authors:** Mohamed K. Y. Soliman, Salem S. Salem

**Affiliations:** Botany and Microbiology Department, Faculty of Science, Al-Azhar University, Nasr City, Cairo Egypt

**Keywords:** SeNPs, PPW, Characterization, Phytochemical study, Antibacterial, Antibiofilm, Gene expression, Antioxidant and anticancer

## Abstract

This research employed *Ananas comosus* (pineapple) peel waste (PPW) extract for producing selenium nanoparticles (SeNPs) using an ecologically feasible way, aimed at various medical uses. Our analysis demonstrated that the PPWextract was a significant supplier of several important phytochemicals. The synthesized SeNPs were comprehensively characterized via XRD, FTIR, SEM, EDX, UV-Vis, and HRTEM which exhibiting a spherical shape with dimensions between 33 and 73 nm. Additional experimental assessments of SeNPs were carried out to ascertain their suitability for usage in biology applications. The findings suggest that obtained SeNPs may effectively combat multiple bacteria, including *S. aureus*,* E. coli*,* B. subtilis*,* E. faecalis*, and *K. pneumonia.* Additionally, SeNPs exhibited antibiofilm capacity for both MRSA and *E. coli* with inhibition reported to be 64.8% and 54.4% at 100 µg/mL respectively. In the range of 62.5–31.25 µg/mL SeNPs reduced expression of two essential genes required for *S. aureus* to generate biofilms, cna (0.9 fold change), and quorum sensing gene LuxS of *E. coli* (4.2 folds of control to 3.4 folds of treated) in comparison to the RecA gene. The antioxidant capacity of SeNPs reported an IC_50_ value of 98.3 µg/mL. The formed SeNPs demonstrated anticancer efficacy in combating the HepG2 malignant cell line, with an IC_50_ value of 113.02 µg/mL.

## Introduction

Nanotechnology is anticipated to be the cornerstone of the next industrial revolution, significantly altering economic systems and global power relations [1, 2]. This sector is very useful in many areas, such as biomedicine, agriculture, wastewater treatment, prevention of infections, and industry [3–7]. The ecologically friendly method of producing the nanoparticle presents a chance enabling its safe application in the medical domain [8–11].

The process of bio-reduction of nanoparticles made of metal to their elemental forms, known as nanoparticle biogenesis, is initiated by functional molecules like amines among alkanes, which are found in large quantities in metabolites such as terpenoids, alkaloids, steroid, and flavonoids which play a significant role in the reduction prcosess to convert metal element to nanoparticles [12, 13]. Due to its outstanding bioactive qualities as well as minimal toxic effects, selenium has lately been employed as a form of nanoparticles. Selenium is a vital mineral that impacts human health and nutritional requirements [14]. Most of the biological systems of the living organisms contain the trace element selenium (Se), which is essential for the functioning of an antioxidant enzyme [15]. Despite the need to stabilize or reduce representations, naturally occurring Se nanoparticles provide a dependable and eco-friendly approach for producing metalـ/metalloid nanoparticles distinguished by enhanced its biological activity and little toxicity [16, 17].

The utilization of microalgae, plants, naturally existing organisms, and enzymes in the formation of nanostructures is known as “green synthesis,” and is advertised as a low-risk, straightforward, safe, and ecologically beneficial process [8, 18]. There are numerous other biosynthetic routes available; however, because of its simplicity and ease of purification, plant-mediated NP biosynthesis has gained a lot of attention [19]. Recent research has effectively produced SeNPs using a variety of plant sources and materials [20, 21]. Zinc, copper, silver, and selenium nanoparticles have been biosynthesized using zero-value residue from agriculture and related commercial byproducts [22–28]. Some of the primary wastes and associated byproducts, including straw, bagasse, leaf, stem, as well as peel, have been utilized [29–31].

 The emerging resistance of microorganisms to antimicrobial medications poses one of the biggest and most significant public health concerns. Drug-resistant bacteria develop novel defense mechanisms against antimicrobial medications, leading to their development and dissemination [32]. Unfortunately, the evolution of pathogens outpaces the ability of current antibiotic-based therapy, leaving the future uncertain. By 2050, Antimicrobial resistance (AMR) will kill over 10 million people globally and cost more than $100 trillion [33]. Furthermore, microbial biofilms are vital in several diseases, and biofilm-associated characteristics may markedly enhance the prevalence of antibiotic-resistant bacteria within microbial populations [34]. The bacterial biofilm matrix [35], which can function as a barrier to movement, may impede antibacterial medications and immune system effectors. Additionally, food shortages or the development of a persistent but nongrowing phenotype that enables microbial cells to adapt effectively to outside stresses like antibiotic treatment can cause bacteria to evolve into exceedingly resistant to antibiotics [36]. About 40% people throughout the industrialized and underdeveloped nations suffer from cancer, making it one of the deadliest and most crippling diseases [37]. SeNPs demonstrate many biological actions, including antibacterial, antioxidant, and anticancer effects [38, 39]. Many researches have reported that SeNPs provide defense against infections caused by pathogenic bacteria in humans, including *E. faecalis*, *B. cereus*, *L. monocytogenes*, *S. agalactiae*, and *S. aureus * [40– 42]. This scenario demands a quick response and a more innovative approach to the creation of new, safe, and effective antibacterial medications utilizing nanotechnology.

**Scheme 1:**
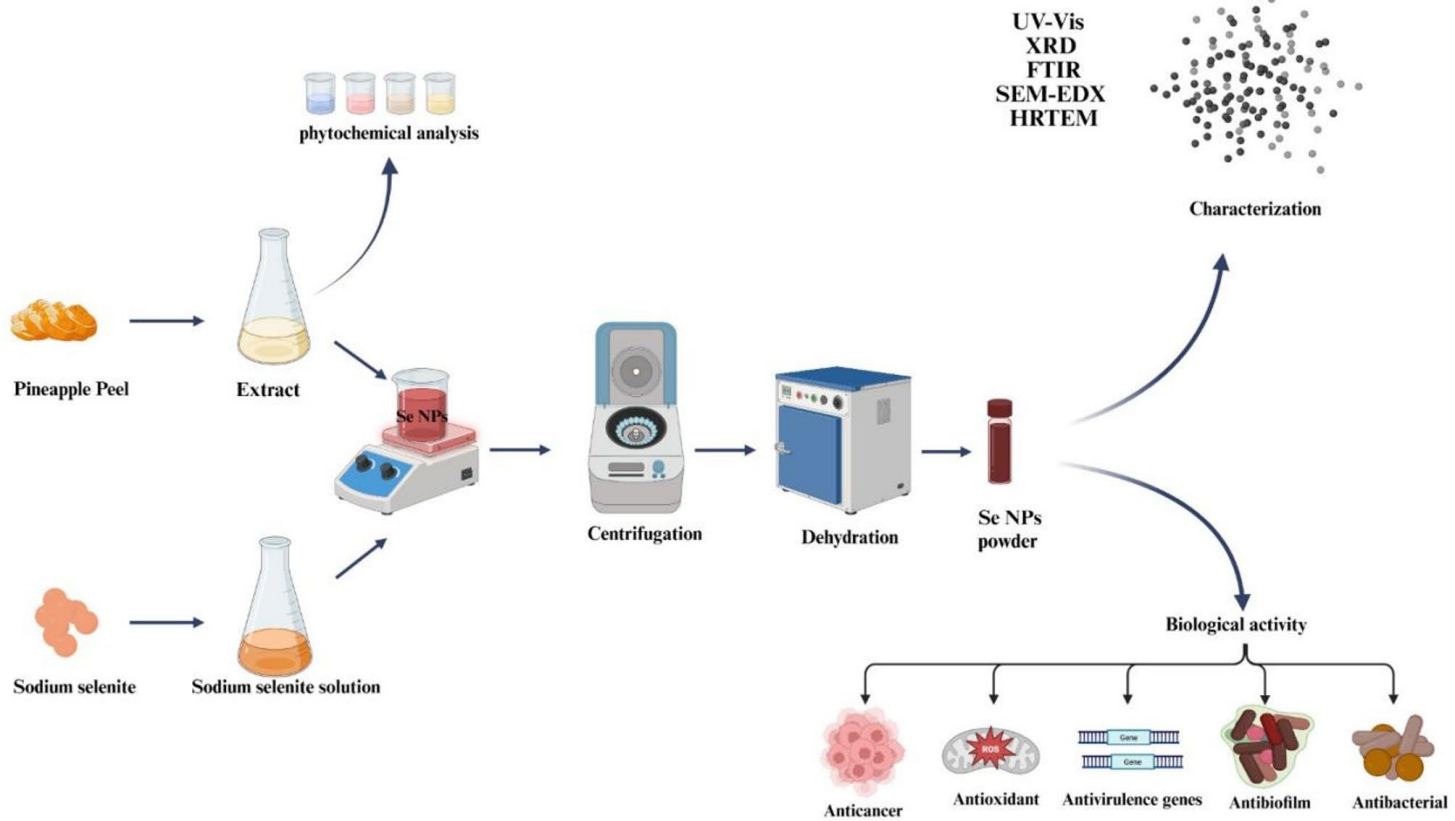
Schematic diagram of the bio fabricated SeNPs via PPW extract and their biological potential

The present work reports an efficient and rapid biosynthesis of SeNPs was achieved by employing pineapple peel extract for the first time, which appears to be an economical, environmentally benign, and green approach. The produced SeNPswas then analyzed using X-ray diffraction (XRD), Fourier transform infrared (FTIR), scanning electron microscope-energy dispersive X-ray spectroscopy (SEM-EDX) V-Visible spectrophotometry, and High-Resolution Transmission Electron Microscopy (HRTEM). The antibacterial, antibiofilm, antioxidant, and anticancer properties of biosynthesized selenium nanoparticles and their impact on the expression of the two essential genes related to biofilm creation and quorum sensing in *S. aureus* and *E. coli* were also investigated (Scheme 1). Taking everything into account, it has been demonstrated that the phytochemical method of SeNPs production is a workable approach for medical applications.

## Materials and methods

### Collection of plant materials

The fresh pineapples were purchased from the neighborhood markets at Qalyube city, Qalyubia governorate, Egypt and thoroughly cleaned by sterile distilled water. The peels were subsequently removed, cut into pieces and washed to remove any remaining dust, dirt, or debris. The cut peels were dried using hot air oven set at 40^°^C for 6–7 h each day for a period of approximately four to five days inorder to eliminate the moisture. Post drying, the dried peels were grounded to a fine powder employing a mixer grinder and stored till further use.

### Preparation of pineapple Peel extract

The extract of pineapple peel waste was prepared by following procedure of Lourenço [43] with minor adjustments. About 10 g dried peel powder was added in 250 mL DDW and heated on a heating mantle for approximately 45 min at 70–80^°^C. The resulting solution was allowed to cool and purified by using vacuum pump filtration. Following filteration, the filtrate was then subjected to centrifugation for 15 min at room temperature at 16,000 rpm. Post centrifugation, the resulting supernatant was refrigerated and stored till further use.

### Qualitative phytochemical analysis

Consistent with previous studies, we performed phytochemical profiling across sample to screen for ingredients components [44–46].

### Quantitative determination of phytochemicals

#### Total phenolic acids

The total amount of phenolic acids in pineapple peel extract was measured using the Folin-Ciocaleau technique as described by Chandra et al. [47]. In the preparation of plant aliquot and standard, the following steps were accounted: A solution was made by mixing 0.2 mL of folin-Ciocalteau reagent (0.5 M) with 0.6 mL of purified water. After 15 min of vigorous shaking, an 8% w/v Na_2_CO_3_ solution (1.0 mL) was added to the mixture and diluting the reaction mixture to a volume of 3.0 mL, and incubated for 30 min in the dark. Absorbance of the characteristic deep blue solution was measured at 760 nm wavelength with triplicate verification. Total phenolic compounds were calculated using the linear regression equation Y = 0.003x + 0.0239 (R² = 0.9942) derived from gallic acid standard calibration. The gallic acid concentrations in methanol were 40, 80, 120, 160, and 200 µg/mL. Using the formula CV/m, the result was presented as gallic acid equivalent [47].

#### Total tannins

The total tannin content was determined using the Folin-Denis spectrophotometric method [48] with minor modifications. Briefly, about 500 µL of sample and 500 µL of deionized water (D·H₂O) were mixed with 0.1 g of polyvinylpolypyrrolidone (PVPP) in a cold environment to bind tannins. The mixture was incubated at 4 °C for 4 h, then centrifuged (10 min) to isolate non-tannin phenolics in the supernatant. Approximately, 100 µL of the non-tannin phenolic supernatant was reacted with 0.5 mL Folin-Ciocalteu reagent (1 N) and diluted to 1000 µL with D·H₂O. After 5 min incubation (room temperature), 2.5 mL of 5% Na₂CO₃ was added to each tube (including blanks). Then, the tubes were vortexed, incubated in darkness (40 min, room temperature), and absorbance was measured at 725 nm against a tannic acid standard curve (Y = 0.0086X + 0.0233, R² = 0.9926). The tannins content of the extract was quantified using the subsequent calculation:

$$\eqalign{ {\rm{Total}}\,{\rm{tannins}}\left( {\rm{g}} \right)&{\rm{ = Total}}\,{\rm{phenolics}}\,{\rm{content}}\left( {\rm{g}} \right) \cr & {\rm{ - non - tannin}}\,{\rm{phenolics}}\,{\rm{content}}\left( {\rm{g}} \right) \cr} $$

All analyses were performed in triplicate.

#### Total flavonoids

The total flavonoid content (TFC) of the pineaple peel extract was determined using a modified aluminum chloride (AlCl₃) colorimetric assay [49], based on the formation of a flavonoid-aluminum complex with characteristic yellowish-orange coloration. Firstly, 2 mL of distilled water, 0.15 mL of 5% sodium nitrite (NaNO₂), and plant extract were mixed and incubated for 5 min at room temperature. Followed by, 0.15 mL of 10% AlCl₃ and 1 mL of 4% NaOH were added sequentially, followed by vortexing. The mixture was incubated at 40 °C for 15 min to develop the chromogenic complex. A quercetin standard curve (varying concentrations) was prepared identically for calibration. Absorbance was measured at 510 nm and TFC was calculated using the linear regression equation:

$$\eqalign{ & y = 0.0034X + 0.0135\left( {R2 = 0.9971} \right) \cr & y = 0.0034X + 0.0135\left( {R2 = 0.9971} \right) \cr} $$

Results were expressed as milligrams quercetin equivalents per gram of extract (Q eq/g), derived from the CV/m formula (consistent with phenolic quantification [49]).

#### Total flavonols

The flavonols content in pineaple peel extract was determined using the aluminum chloride (AlCl₃) colorimetric method as described by Miliauskas et al. [50]. The analytical procedure involved mixing 2 mL of AlCl₃ solution with 6 mL of sodium acetate (CH₃COONa) buffer, followed by incubation at 20 °C for 2 h to allow for complete complex formation between the flavonols and aluminum ions. After incubation, the absorbance of the samples was measured at 440 nm using a UV-Vis spectrophotometer. A standard curve was prepared using rutin as the reference compound, following the same protocol. All samples were analyzed in triplicate to ensure method reproducibility. The total flavonol content was calculated and expressed as milligrams of rutin equivalents per gram of dry weight (mg RT eq/g DW), with results presented as mean values ± standard deviation (*n* = 3).

#### Total alkaloids

The total alkaloid content in pineaple peel extract was quantified according to the gravimetric method described by Harborne [51]. In this procedure, 1 g of dried plant powder was macerated with a 4:1 (v/v) mixture of glacial acetic acid and 70% ethanol. The extraction was allowed to proceed for a minimum of 6 h to ensure complete alkaloid dissolution, after which the mixture was filtered to remove particulate matter. Alkaloids were then precipitated from the filtrate by gradual addition of concentrated ammonia solution. The resulting precipitate was collected by filtration through pre-weighed filter paper and subsequently dried to constant weight. The alkaloid content was calculated based on the weight difference of the filter paper before and after precipitation. Final results were expressed as milligrams of alkaloids per 100 g of dry weight (mg/100 g DW), providing a standardized measure of alkaloid content in the plant material.

#### Total saponins

The saponin content was determined via ethanol extraction and solvent partitioning [52]. The Sample (5 g) was extracted twice with 100 mL of 20% aqueous ethanol at 55 °C for 4 h, with continuous stirring. The combined extracts were concentrated to about 40 mL, then sequentially washed with diethyl ether and extracted with n-butyl alcohol. The n-butyl alcohol phase was purified with 5% NaCl solution, evaporated to dryness, and oven-dried to constant weight. Saponin concentration was calculated gravimetrically and expressed as mg/100 g dry weight.

#### Total steroids

For steroid analysis, sample (5 g) was hydrolyzed by refluxing in 50 mL of HCl for 30 min [51]. The acid-hydrolyzed mixture was filtered (Whatman filter paper) and partitioned with ethyl acetate (1:1 v/v) in a separatory funnel. The ethyl acetate layer was collected, evaporated at 100 °C, and resuspended in hot amyl alcohol to extract steroids, yielding a characteristic turbid solution. The steroid fraction was filtered, desiccated, and weighed to determine concentration gravimetrically. Results were expressed as mg steroids per 100 g dry weight.

### Green synthesis of SeNPs

The biosynthesis of SeNPs was carried out using pineaple peel extract as a reducing and stabilizing agent. The extract (60 mL) was placed in a thermostatically controlled beaker on a magnetic stirrer and maintained at 60 °C with continuous stirring (250 rpm). Under constant agitation, 8 mL of aqueous sodium selenite solution (25 mM) was added dropwise using a micropipette. The reaction mixture was allowed to stir for an additional 2 h to ensure complete reduction. Following the reaction, the solution was refrigerated at 2–8 °C for 48 h to facilitate nanoparticle maturation. The resulting SeNPs were then isolated by centrifugation at 16,000 rpm (4 °C) for 15 min. After discarding the supernatant, the pellet was collected and dried in a hot-air oven at 200 °C for subsequent characterization and analysis [53, 54].

### Optimization assessment of SeNPs biosynthesis

Throughout optimization trials, the parameters associated with the filtrate and its primary nanoparticle component, sodium selenite, were optimized. The effects of varying durations between the filtrate and precursor solutions (0.5, 1.0, 1.5, 2.0, and 2.5 h), precursor concentrations (5, 10, 15, 20, 25, and 30 mM), pH value (6, 7, 8, 9, and 10), and temperature (30, 40, 50, 60, and 70 °C) were subsequently assessed using the one variable at a time (OVAT) method [8, 55, 56].

### SeNPs characterization

The UV–visible spectrophotometry (JASCO ـV 630, Hachioji, Japan) was used to detect prominent peaks of absorption corresponding to surface plasmonic resonance of the synthesized SeNPs over the visible UV spectrum, which spans from 200 ـ 800 nm. The transmission electron ـmicroscope (JEOLــ2100, Akishima, Japan) was used to investigate the size and shape of SeNPs. The selenium nanoparticles’ components were investigated for assessment of functional groups by Fourier Transform Infrared spectroscopy (FT-IR) (JASCO, FT/IR-6100). The synthesized SeNPs were compacted into discs and amalgamated with potassium bromide (KBr) for analysis. The FT-IR spectra was scanned in the range of 400–4000 cm^− 1^. Additionally, X-ray diffraction (XRD; Philips, Netherlands) was used to ascertain the crystalline nature of SeNPs. XRD patterns were oriented inversely with respect to a nickel ـfiltered Cu ــKα electromagnetic radiation source functioning at 40 kv and 30 mA. The crystalline form of generated SeNPs was analyzed for duration of two hours, within the temperature range of 10^°^ to 80^°^. Additionally, the morphology and dimensions of the synthesized SeNPs were evaluated by SEM analysis (SEM, ZEiSS, EVO-MA 10, Oberkochen, Germany). The constituents of nanoparticles were analyzed for elemental composition, purity, and dispersion using EDXــBRUKER, NanoــGmbH, (D-12489, Mــ410) Germany [57, 58].

### Antibacterial assessment

The efficacy of SeNPs was tested against five pathogenic bacteria: *B. subtilis*, *S. aureus*,* K. pneumoniae*, *E. faecalis*, and *E. coli*. The agar-well dispersion technique was used for the investigation as follows: The bacterial suspension (10 µl) was uniformly spread onto the sterile MH agar medium, after cultivation in MH broth [59]. About 7 mm well was created on each plate with a sterile corkـborer and subsequently 100 µL SeNPs (1000 µg/mL) were added to the wells followed by incubation at 37^°^C for 24 h. The size of zones of inhibition were evaluated [60]. The test was performed in triplicates to ensure accuracy.

Using the broth microdilution technique, the minimum inhibitory concentration (MIC) of the SeNPs against pathogenic strains were evaluated. This procedure assisted in determining the MIC of the biosynthesized SeNPs. Selenium NPs were serially diluted twice, and 5 µL microbial culture equivalent to 0.5 McFarland was added to the prepared SeNPs. The resulting mixtures were incubated for 24 h at 37^°^C. Post incubation, 30 µL resazurin dye was added to all the wells and incubated under dark conditions. Upon incubation, the hue transitions from blue to pink, was observed demonstrating bacterial activity. Triplicates readings were averaged to determine the MIC [60, 61].

### Anti-biofilm assessment

The anti-biofilm efficacy of SeNPs against clinical isolates of *S. aureus* and *E. coli* was assessed using a modified microtiter plate (MTP) assay [62]. Briefly, SeNPs at varying concentrations (100, 50, 25, 12.5, 6.25 and 3.12 µg/mL) were co-incubated with bacterial inoculum (0.5 McFarland standard, 1:100 dilution in TSB + 1% glucose) in 96-well plates at 37 °C for 48 h. After incubation, planktonic cells were gently removed, and adherent biofilms were washed with PBS (pH 7.4), fixed with 95% methanol (200 µL/well), and stained with 0.3% crystal violet (CV) for 15–20 min at room temperature. Excess stain was removed by distilled water washing, and biofilm-bound CV was solubilized with 30% acetic acid (200 µL/well). Absorbance was measured at 540 nm using a Tecan Infinite^®^ M200 microplate reader [63]. with untreated wells serving as controls.

### RNA isolation and cDNA synthesis

#### RNA isolation

Total RNA was isolated from both bacterial strains using TRIzo reagent (Gibco) following the manufacturer’s protocol. The cDNA for the sense strands was generated using the RT ــPCR Kit (Clontech, Palo Alto, USA).

#### Traditional PCR amplification for reverse transcription

The primers listed in Table 1 were used to amplify the Cna gene of *S. aureus* in addition to the LuxS gene of *E. coli* using a PCR combination in 50 µL experiments. RecA, the reference housekeeper gene, was amplified using primers indicated in Table 1. The primer-BLAST tool on the NCBI website was used to design these primers.

The process is described as follows: The experiment included 29 rounds of 35 s of 93^°^C, 45 s at 50^°^C, one minute at 45^°^C, and 6-minute elongation at 72^°^C, preceded by a 4 min at 93^°^C. The agarose gel was prepared using a 1% TAE buffer containing 40 mM Tris-Acetate and 1 mM Na-acetic acid (EDTA) at pH 7.6. The gel was subjected to electrophoresis, stained with ethidium bromide (0.5 mg/mL), and then examined under UV light. A standard length DNA ladder (GeneRulerTM 100 bp DNA Ladder, MBI Fermentas, Vilnius, Lithuania) was used to determine the size.

Conditions: Semi-quantitative PCR was conducted on cDNA of the *S. aureus* strain using the primers specified in Table 1. The PCR mixture consisted of: 12.5 µL containing 2× Quantitech SYBR^®^ Green RT Mix, 1 µL each of both reverse and forward primers at a concentration of 25 pmol, 1 µL of 50 ng cDNA, and 9.25 µL of RNase-free water. Table 1 had two primers exclusive to the fnbA gene, whereas Table 2 included two primers exclusive to the Cna gene. In real-time PCR protocols, the first denaturation step occurs over 40 cycles of 15 s at 93^°^C for a duration of 10 min, 35 s of annealing at 45^°^C and 30 s of extension at 72^°^C.

**Table 1:** Primer for detection of the can, LuxS genes in *S. aureus* and *E. coli* strains

Primers	Sequence (5’->3’)	strand	Length	Tm	GC%	Self-3’ complementarity
LuXS-FW	ATCCATACCCTGGAGCACCT	Plus	20	60	55	2
LuxS-RW	GCCACACTGGTAGACGTTCA	Minus	20	60	55	2
cna-FW	AAGGTGAACAGGTGGGTCAA	Plus	20	59	50	0
cna-RW	CACTACTTGTTCCCGCTTCA	Minus	20	57	50	1
RecA1-FW	GCCCTAATTGGTCCAGGCG	Plus	19	45	44	0
RecA1-RW	ACAACGGCGTTCTCTCCTAT	Minus	20	45	44	0

### Antioxidant efficacy

By effectively neutralizing DPPH (2,2 ــdiphenyl-1 ــpicrylhydrazyl) radicals, the ability of generated SeNPs to function as antioxidants was assessed throughout a range of concentration (15.62–1000 µg/mL). A 1 mM DPPH radical solution was prepared in 95% ethanol. For the assay, 200 µL of each concentration of biosynthesized SeNPs was mixed with 800 µL of the DPPH solution. The reaction mixture was vortexed briefly and incubated in darkness at 25 °C for 30 min. Following incubation, samples were centrifuged at 13,000 (rpm) for 5 min to pellet any particulate matter [64]. The supernatant absorbance was then measured at 517 nm to determine radical scavenging activityAscorbic acid was employed as a standard, while the absorbance value was measured at 517 nm in relation to a blank.

### Anticancer and cytotoxicity assessment

The American Type - Culture Collection (ATCC) provided the hepatocellular adenocarcinoma cells (HepG2) and normal foreskin cells (HFP4) used in the different cell cultures. The 3 ـ(4,5-dimethylthiazol ـ2-yl)-2,5 ـdiphenyltetrazolium bromide (MTT) test was used to evaluate the cytotoxic properties of SeNPs. In order to create a thick monolayer cultivation of cells in microtiter plate (MTP) for 24 h at 37^◦^C. One million cells for each well were used as the inoculation density for each well. The growth fluids were discarded after a confluent cell layer had formed, and the cells were repeatedly cleaned with washing liquid to eliminate any remaining monolayer. A medium for proliferation (RPMI medium) supplemented with 2% bovine fetal serum was employed to include the biosynthesized SeNPs by two-fold serial dilutions. Three wells were assigned as controls and were provided alone with the maintenance medium during the experiment. Each well received 100 µl of the following concentrations: 15.62–500 µg/mL. After incubation, the cells were assessed individually for indicators of cytotoxicity, such as rounded off, shrinking, granulating, or a partial or total breakdown of the monolayer film. The dish was positioned on a shaking device and stirred for an additional 5 min at approximately 150 rpm for thorough mixing of MTT and medium. The prepared plate was then subsequently maintained for 4 h at 37^°^C containing 5% CO_2_ to ensure the full metabolism of MTT. The medium was discarded and subsequently 0.2 mL DMSO was used to re-dissolve formazan, a metabolite of MTT. The mixture was agitated for 5 min at 150 rpm to ensure the thorough dissolution and integration of the formazan with the solvent. The absorbance at 560 nm was measured after eliminating any disturbances at 620 nm [65, 66].

### Statistical analysis

Statistical studies were determined via utilizing the Minitab 18 program. One-way ANOVA was applied, with a significance value of *p* < 0.05. All experimental assays were conducted in triplicates.

## Results and discussion

### Phytochemicals screening of pineapple peel extract

Phytochemicals are used in both conventional and contemporary medical systems and are essential for the treatment of many illnesses. The qualitative chemical tests provide a variety of insights on the types of phytochemical components found in the crude medication. The presence of glycosides, alkaloids, tannin, saponins, flavonoids, steroids, phenols, diterpenes, quinones, proteins, amino acids, and carbohydrates, had been identified by making chemical tests as preliminary phytochemical screening of *Ananas comosus* (pineaple) dried peel as illustrated in Table 2. While anthraquinones, and cardiac glycosides were found to be absent. These results are consistent with [67, 68].

**Table 2:** Preliminary phytochemical screening of *Ananas comosus*

Phytochemicals	*Ananas comosus*
Flavonoids	**+**
Cardiac glycosides	**-**
Alkaloids	**+**
Steroids	**+**
Saponins	**+**
Phenols	**+**
Anthraquinones	**-**
Glycosides	**+**
Diterpenes	**+**
Quinones	**+**
Tannins	**+**
Proteins	**+**
Amino acids	**+**
Carbohydrates	**+**

+ (presence of phytoconstituents); - (absence of phytoconstituents)

Phytochemicals play essential structural and protective roles in plants, enhancing their resistance to diseases, predators, and environmental stressors. These compounds are synthesized through secondary metabolic pathways in response to adverse conditions such as UV radiation, temperature extremes, acidity, and heavy metal exposure. Among the most significant phytochemicals are phenolic compounds, which contribute to the plant’s physiological and morphological resilience [69–71]. Beyond their role in plants, phytochemicals exhibit numerous bioactive properties when consumed in foods like fruits and vegetables. Research highlights their antioxidant [72], anti-inflammatory, antithrombotic, antiallergic, antitumor, cardio-protective [73] and antimicrobial effects [74] underscoring their potential in promoting human health. The total amounts of flavonoids, phenolic acids, and tannins in *Ananas comosus* were reported as 32.67 ± 0.66 mg QE per gram of dry weight, 14.88 ± 0.39 mg RTE per gram of dry weight, 42.02 ± 0.51 mg GAE per gram of dry weight, and 29.92 ± 0.43 mg TAE per gram of dry weight, respectively (Table 3). This is similar to the results of Rolim et al. [75]. The total alkaloid, saponin, and steroid content in the current study were reported to be 0.59 ± 0.07 mg /100 g Dry weight, 2.12 ± 0.05 mg /100 g Dry weight, and 0.71 ± 0.07 mg /100 g Dry weight, respectively.

**Table 3:** Quantitative phytochemical analysis of *Ananas comosus*

Parameters	*Ananas comosus*
Total flavonoids (mg QE /g Dry -weight)	32.67 ± 0.66
Total flavonols (mg RTE /g Dry -weight)	14.88 ± 0.39
Total phenolic acids (mg GAE /g Dry -weight)	42.02 ± 0.51
Total tannins (mg TAE /g Dry -weight)	29.92 ± 0.43
Total alkaloids (mg /100 g Dry -weight)	0.59 ± 0.07
Total saponins (mg /100 g Dry -weight)	2.12 ± 0.05
Total steroids (mg /100 g Dry -weight)	0.71 ± 0.07

### Green biosynthesis of SeNPs using pineapple peel waste

**Fig. 1:**
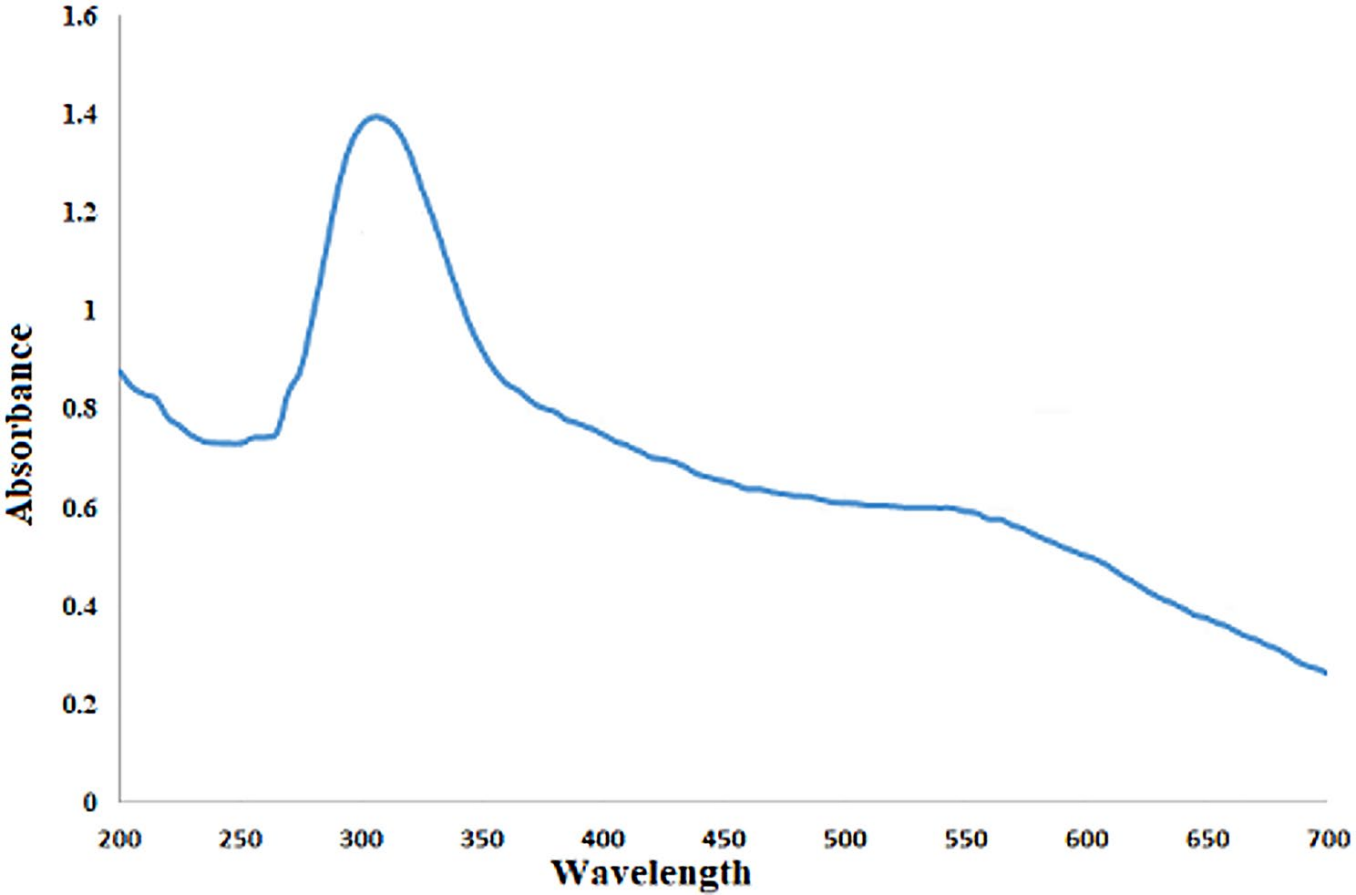
Ultraviolet-visible spectra of SeNPs generated from PPW extract

Biological, nutritional, and nanotechnological fields place a high priority on plant resources because of their perceived sustainability, lack of toxicity, and lack of negative environmental impact. As an eco-friendly substitute for traditional chemical and physical methods, plant-based nanoparticle synthesis has become increasingly popular in the last decades [76, 77]. The presence of reddish-colored solution due to PPW extract-selenite interaction demonstrated the synthesis of SeNPs, as the components in the extract were able to decrease the selenite ions and convert them to SeNPs. Sustainable SeNPs were produced by using the PPW as a reducing and stabilizing agent. The PPW bio reduction of Na_2_SeO_3_ to SeNPs was confirmed by a gradual change in solution color from light yellow to dark red, which signifies the synthesis of SeNPs. A comparable change in hue from yellow to reddish-brown validated the development of SeNPs. A notable peak at 305 nm was seen in the ultraviolet (UV) examination of PPW-synthesized SeNPs, as shown in Fig. 1. The generation of SeNPs could be confirmed using the UV-visible spectrophotometer due to the SeNPs’ surface-plasmon resonance (SPR) signal, which could be represented as a broad wavelength of stimulation (λ _max_) ranging from 270 to 400 nm [78]. The resultant sustainable production of SeNPs is simple, eco-friendly, and economical; the NPs produced are safe for the environment and exhibit excellent stability. Many methods have been described for synthesizing SeNPs using water-based extracts of different plant parts [79, 80].

**Fig. 2:**
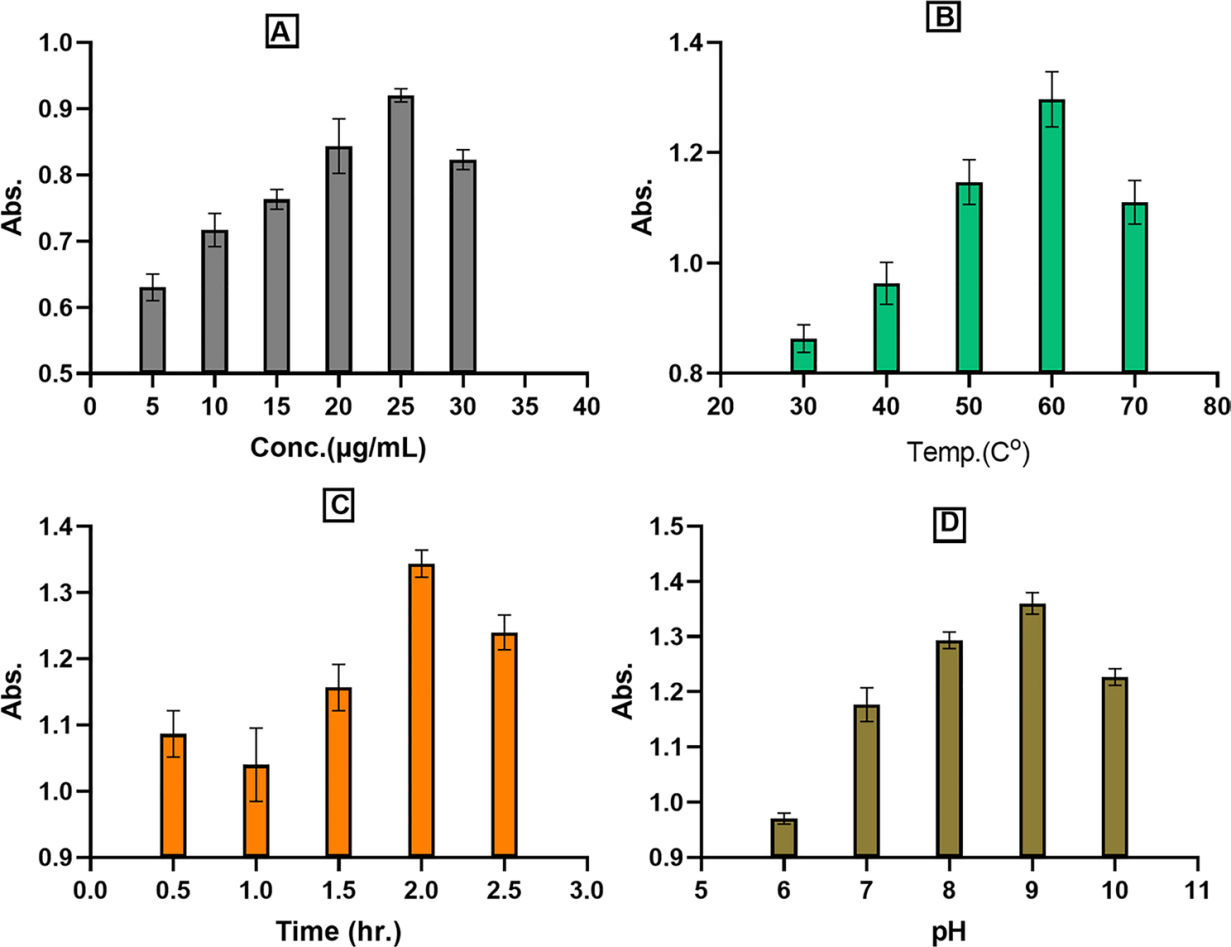
Different factors influence the productivity of SeNPs

As illustrated in Fig. 2, the influence of varying sodium selenite precursor concentrations indicated that the optimal concentration for maximum SeNPs formation was 25 mM, as described by UV–visible spectra (Fig. 2A). In addition to at lower concentrations < 25 mM may lead to incomplete reduction, while higher concentrations > 25 mM can overwhelm the reducing capacity of the extract, resulting in polydisperse particles or residual toxicity [56].*Bacillus subtilis* L11 achieved 100% conversion at 4 mM selenite, whereas plant-mediated synthesis as *Prunus persica* required 10–20 mM for optimal yield [81].The peak productivity of SeNPs was achieved at 60 °C in comparison to other temperatures, as illustrated in (Fig. 2B). In other research, *Trichoderma* sp. showed peak SeNP production at 37 °C, while citrus-based synthesis required 60–80 °C for rapid reduction [82, 83]. The ideal incubation duration for achieving maximum SeNPs production occurred at 2 h, when the filtrate of pineapple peel had been infused with sodium selenite solution, resulting in the highest optical density (Fig. 2C). Shorter durations less than 2 h. may leave unreacted selenite, while longer durations more than 4 h. risk particle aggregation [82]. The impact of varying pH levels on the production of SeNPs was diverse, with the optimal pH identified at 9, corresponding to a wavelength of 305 nm (Fig. 2D). At higher pH, phenolic compounds, flavonoids, and proteins in plant extracts (pineapple peel) exhibit stronger reducing activity, facilitating the efficient conversion of selenite to elemental selenium [82, 84]. Alkaline conditions promote electrostatic repulsion between negatively charged SeNPs (due to carboxylate and hydroxyl groups from biomolecules), preventing aggregation and ensuring colloidal stability [85]. A study using *Trichoderma* sp. found pH 8 optimal for SeNP formation, but plant-based systems (e.g., citrus extracts) often require higher pH (9–10) due to the redox properties of phytochemicals [83]. In the creation of a biotechnological production method for scientifically valuable chemicals, it is essential to optimize incubation temperatures, aeration, pH, oxygen levels, and media composition to increase growth and yield [86, 87]. An additional rise in the pH as well as temperature resulted in a reduction of biosynthesis. This decline parallels studies of activity inactivation under elevated physical circumstances, including pH and temperature [88, 89].

**Fig. 3:**
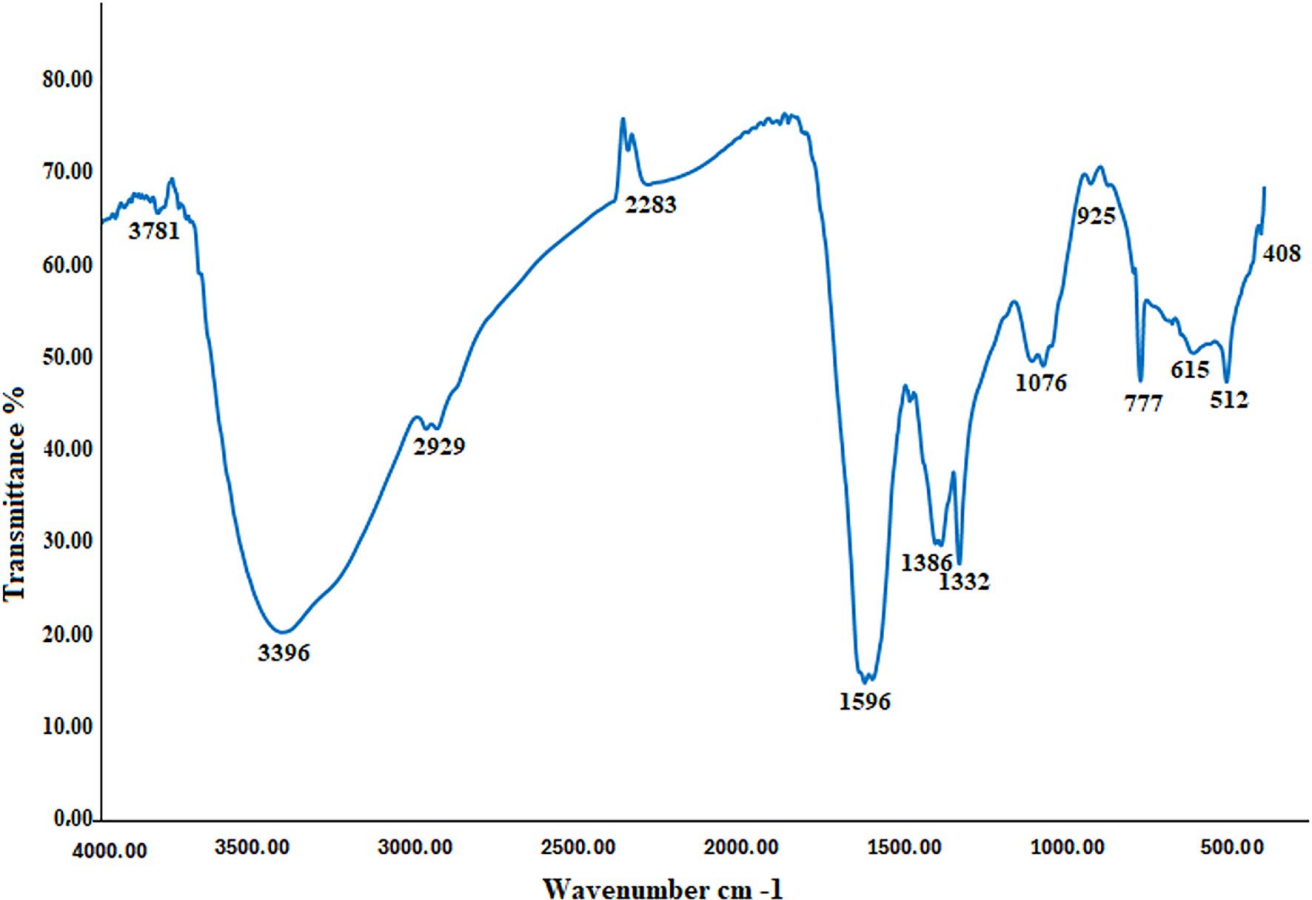
FTIR of SeNPs generated from PPW extract

Furthermore, FT-IR spectroscopy analyses were performed to validate the prospective involvement of PPW extract in the synthesis of SeNPs [90]. FT-IR can identify the surface groups which functions with SeNPs by recording the stimulation of bonds between molecules. The obtained molecular data makes it easy to gain insights into structural alterations in the supporting characteristic functional groups on Se nanoparticle surfaces [91, 92]. The wave numbers at 3781, 3396, 2929, 2283, 1596, 1386, 1332, 1076, 925, 777, 615, 512, and 408 cm^− 1^ showed the relationship of a capping compound from the PPW extract with SeNPs, as illustrated in Fig. 3. At 3781 and 3396 cm^− 1^ is related to O-H stretching vibrations from free hydroxyl groups, often found in phenols or alcohols. This high-frequency region suggests minimal hydrogen bonding, possibly from isolated -OH groups in polyphenols or water molecules [93]. While, C-H asymmetric stretching found in aliphatic chains of fatty acids, terpenes, or other organic compounds in the extract at nearly 2929 cm^− 1^ [94]. However, 2283 cm^− 1^ may indicate C ≡ N stretching or cumulated double bonds N = C = O. Alternatively, it could arise from selenium-related bonds in the nanoparticle capping layer [95]. At 1596 cm⁻¹ notice to C = C aromatic stretching of flavonoids or amide I (C = O), which confirmed polyphenolic or proteinaceous capping agent [96]. At 1386 cm⁻¹ and 1332 cm⁻¹ indication to C–H bending or carboxylate (–COO⁻) in terpenoids or symmetric stretching of carboxylates [97]. These results provide evidence that proteins interacting with SeNPs may help in their stabilization. The functional relevance of proteins depends not only on their size and structure but also on their precise molecular arrangement. Nanoparticles may bind to proteins through residues of cysteine or unbound amine groups [98]. The extract includes proteins that could communicate with the metal-nanoparticles through their inherent amino or carboxyl-groups [79]. Low-frequency modes confirm SeNPs formation due to presence of 615, 512, and 408 cm^− 1^ [99].

**Fig. 4:**
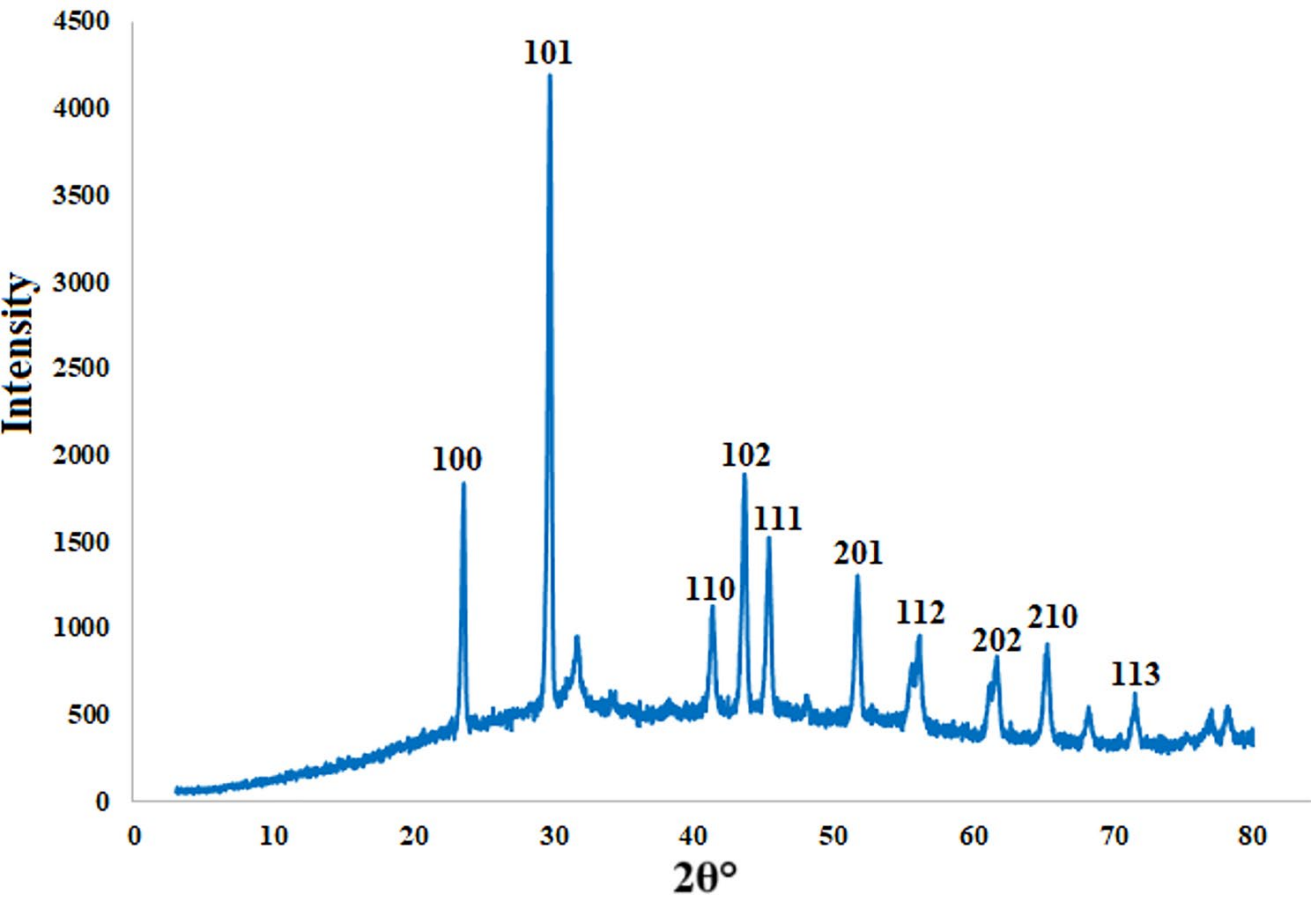
XRD of SeNPs generated from PPW extract

An XRD technique was used to investigate the crystal form and phase of the synthesized SeNPs. Figure 4 illustrates the X-ray diffraction pattern of the synthesized selenium nanoparticles. The XRD diffraction peaks of SeNPs show specific patterns at angles of 23.55^°^, 29.73^°^, 41.38^°^, 43.68^°^, 45.42^°^, 51.72^°^, 56.19^°^, 61.7^°^, 65.32^°^, and 71.56^°^, which match Bragg’s reflections at (100), (101), (110), (102), (111), (201), (112), (202), (210), and (113) of hexagonal crystalline selenium. The highest signal for the (101) plane surpassed the others, indicating its dominant orientation. The findings indicated that the synthesized SeNPs consisted of high-purity hexagonal crystallized selenium [100, 101].

**Fig. 5:**
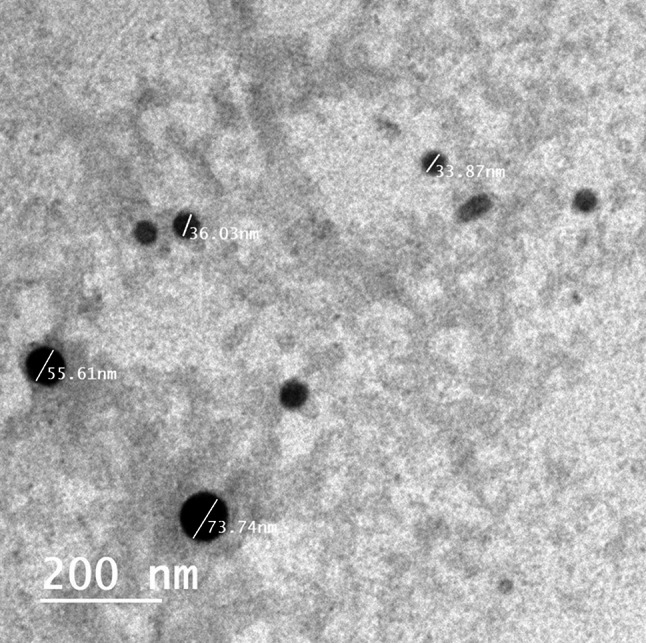
TEM of SeNPs generated from PPW extract

TEM analysis of the colloidal SeNPs demonstrated the formation of polydispersed nanoparticles with dimensions between 33 and 73 nm, as seen in Fig. 5A study by Arumugham et al. [102] reported produced nanomaterial of spherical in shape, measuring 30–60 nm in diameter. Hassanien et al. [103] documented the successful synthesis of SeNPs from *M. oleifera* extract, characterized by their monodispersity, spherical morphology, and size ranging from 23 ـ 35 nanometers. A TEM research verified that the components found in *A. terreus* fungal biomass filtrate (FBF) could be used to create biosynthesised SeNPs with distinctive topologies [104].

**Fig. 6:**
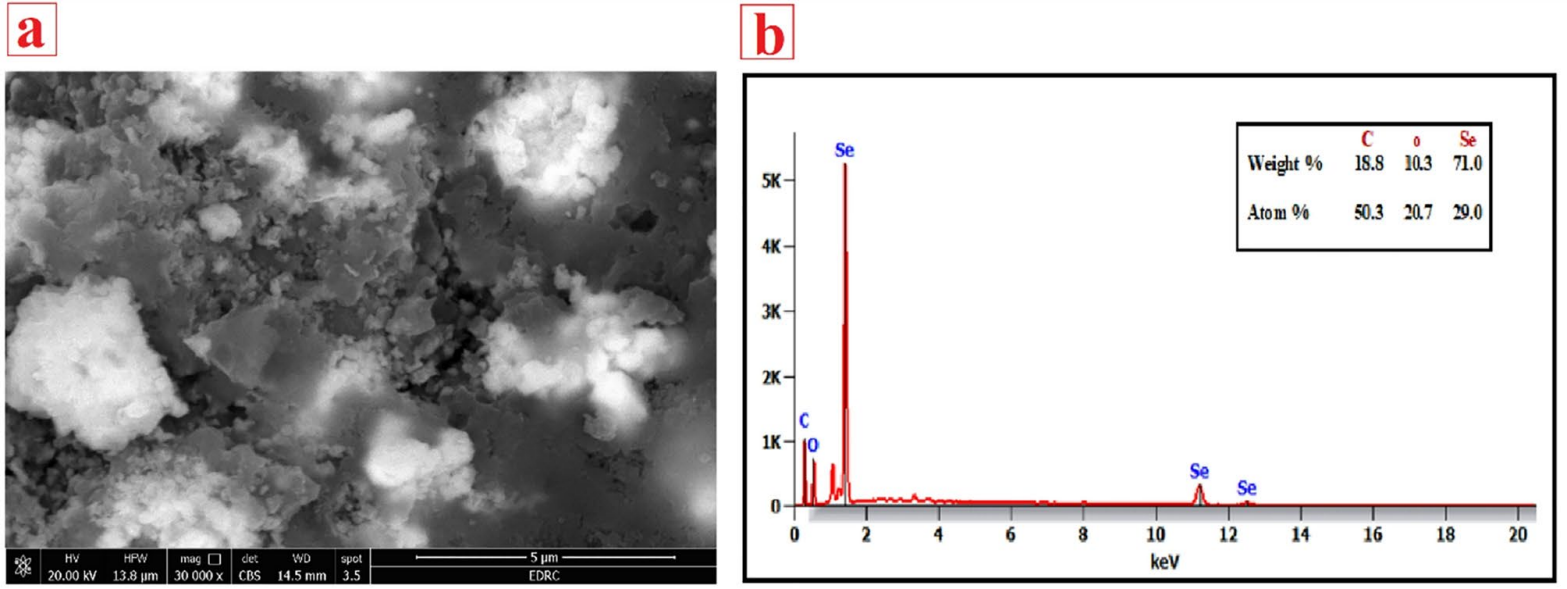
(**a**) SEM image of SeNPs generated from PPW extract (**b**) EDX spectrum of SeNPs generated from PPW extract

SEM was employed to analyze the morphological features of SeNPs. SeNPs exhibited spherical nanomaterials with a smooth outer layer, as seen in Fig. 6a, which is the prevalent morphology of SeNPs corroborated by the research [105]. The scanning electron microscopy indicated that the particles within powder form were somewhat agglomerated. Some SeNPs were discovered to cluster, forming bigger particles. This outcome aligned with the SeNPs acquired in prior research [103]. The EDX analysis of SeNPs verified the existence of the selenium element. The EDX profile in Fig. 6b indicates that the weight fraction of Se element to be 71.0%. The weight proportions of carbon and oxygen were 18.8% and 10.3%, respectively. The greatest atomic proportion was for carbon (50.3%) followed by selenium (29.0%).

### Antibacterial assay

This investigation utilized the agar well diffusion technique to evaluate the antibacterial activity of the biosynthesized SeNPs against a variety of pathogenic bacteria. The SeNPs showed strong antibacterial effects, especially against *K. pneumoniae*,* E. coli*, and *B. subtilis*, with inhibition zones measuring 24.7 ± 0.69, 23.03 ± 0.5, and 20.06 ± 0.35 mm, respectively. *S. aureus* and *E. faecalis*, on the other hand, showed inhibitory zones of 14.86 ± 0.3 and 18.23 ± 0.65 mm, respectively. This outcome demonstrates the produced SeNPs potential as a strong antibacterial drug against a variety of bacteria. We investigated the inhibitory effects of SeNPs at different concentrations (15.62–1000 µg/mL) in order to calculate the MIC. The SeNPs exhibited MIC of 62.5 µg/mL for *B. subtilis* and *E. coli*, whereas the MIC for *S. aureus* and *E. faecalis* was 125 µg/mL. The MIC for *K. pneumoniae* was found to be 31.25 µg/mL, as shown in Table 4.

**Table 4:** Antibacterial activity of SeNPs

No.	Strain name	MIC µg/ml	Diameter of inhibition zone (mm)
1	*S. aureus*	125 ± 0.75	14.86 ± 0.3
2	*B. subtilis*	62.5 ± 0.62	20.06 ± 0.35
3	*E. faecalis*	125 ± 0.82	18.23 ± 0.65
4	*K. pneumoniae*	31.25 ± 0.56	24.7 ± 0.69
5	*E. coli*	62.5 ± 0.49	23.03 ± 0.5

**Fig. 7:**
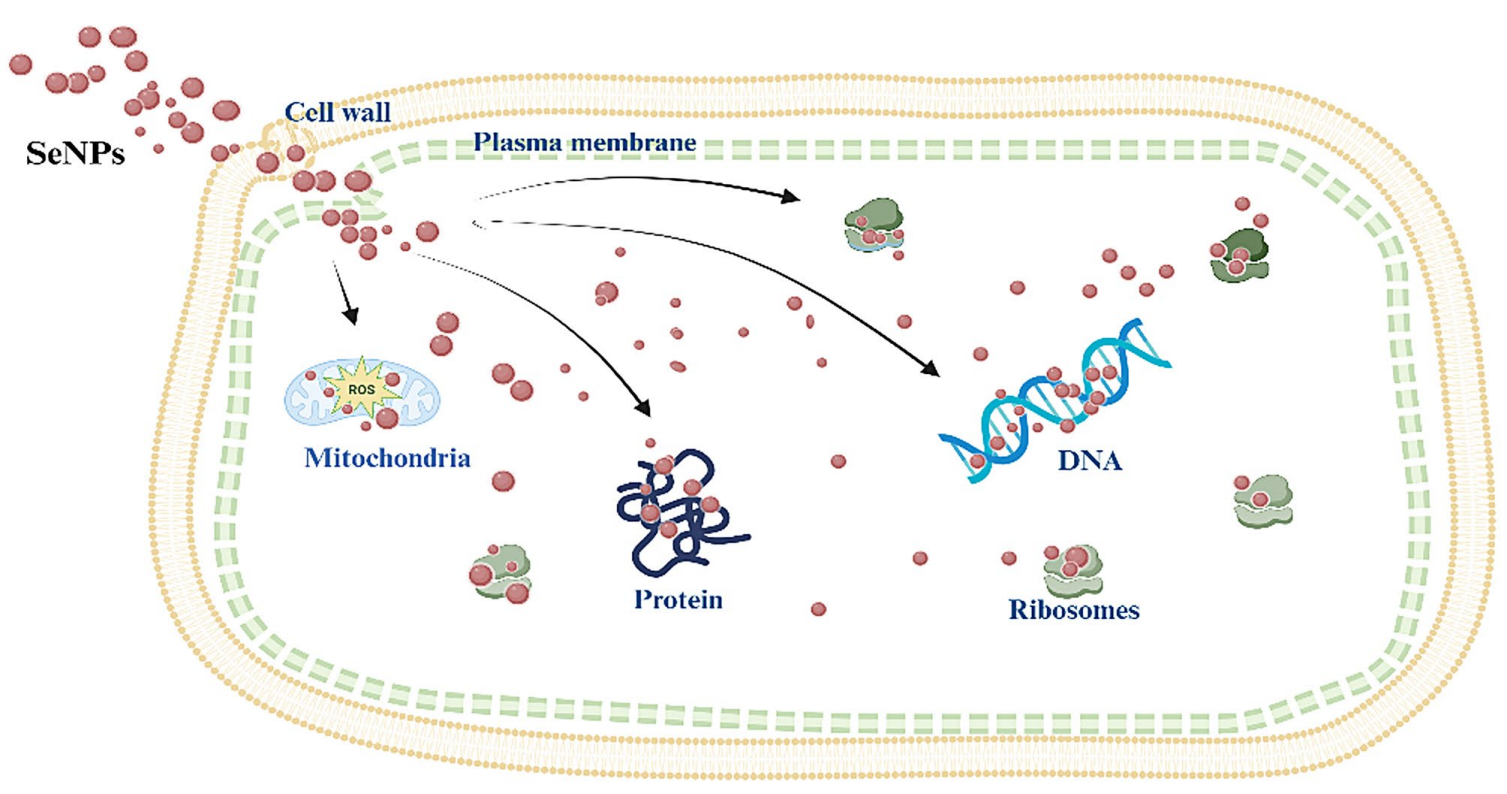
The antibacterial mechanism of SeNPs diagram

*S. aureus*,* Aeromonas hydrophila*, *E. coli*, and *P. aeruginosa* were all susceptible to the SeNPs generated from the polysaccharide found in *Rhizophora mucronata* leaf [106]. At a dose of 100 µg/mL, the antibacterial efficacy of RMLP-SeNPs, SeNPs, Na_2_SeO_3_, as well as a positive control was evaluated [106]. Previous studies have shown that the shape of SeNPs also affects their combativeness towards bacteria; for instance, spherical nanoparticles are taken up by cells more easily as they can better interact with cell membranes compared to larger, elongated particles [107]. In another study, SeNPs produced with sizes that varied from 21.7 to 83.6 nm have been shown to have MICs of 250, 31.25, and 500 µg mL^− 1^ against *E. coli*,* P. aeruginosa*,* B. subtilis*, and *S. aureus*, respectively [108]. In another study, SeNPs synthesized from the water extract of *Annona muricata* fruit were found to effective against different types of bacteria. The analyzed range of the zone of inhibition in case of Gram-positive bacteria ranged between 9.17 ± 0.23 to 23.41 ± 0.50 mm. The maximum zone of inhibition was exhibited by *E. faecalis* (MTCC 439) (23.41 ± 0.50 mm) while the lowest zone of inhibition was reported against *S. aureus* (ATCC 14458) (9.17 ± 0.23 mm). The measured range of the zone of inhibition in Gram-negative bacteria ranged from 4.24 ± 0.31 to 14.35 ± 0.72 mm. *K. pneumoniae* (ATCC 13883) reported the smallest zone of inhibition (4.24 ± 0.31 mm) and *E. coli* (MTCC 41) exhibited the greatest zone of inhibition (14.35 ± 0.72 mm) [109]. SeNPs, of size < 100 nm, synthesized by *S. maltophilia* SeITE02 documented stronger antibacterial effects against *S. aureus*,* P. aeruginosa*, and *E. coli* compared to those between 100 and 400 nm in size [41]. SeNPs exhibits powerful antibacterial effects, and can release selenium ions that damage bacteria, making them a good option for treating MR bacteria [110]. The generation of reactive- oxygen species (ROS), which undermines the integrity of plasma membranes and selective permeability, is likely the mechanism behind the inhibitory effect of SeNPs. Moreover, by disrupting the cell -wall, adhering to the membrane, infiltrating the cytoplasm, and modifying or mutating DNA- replication, metabolic pathways, protein manufacturing, and interactions with thiol-containing or sulfur hydroxide groups in amino acids and proteins—resulting in denatured state and cellular demise—these synthesized SeNPs may exert a favorable impact on bacteria (Fig. 7) [111].

### Antibiofilm of SeNPs

**Fig. 8:**
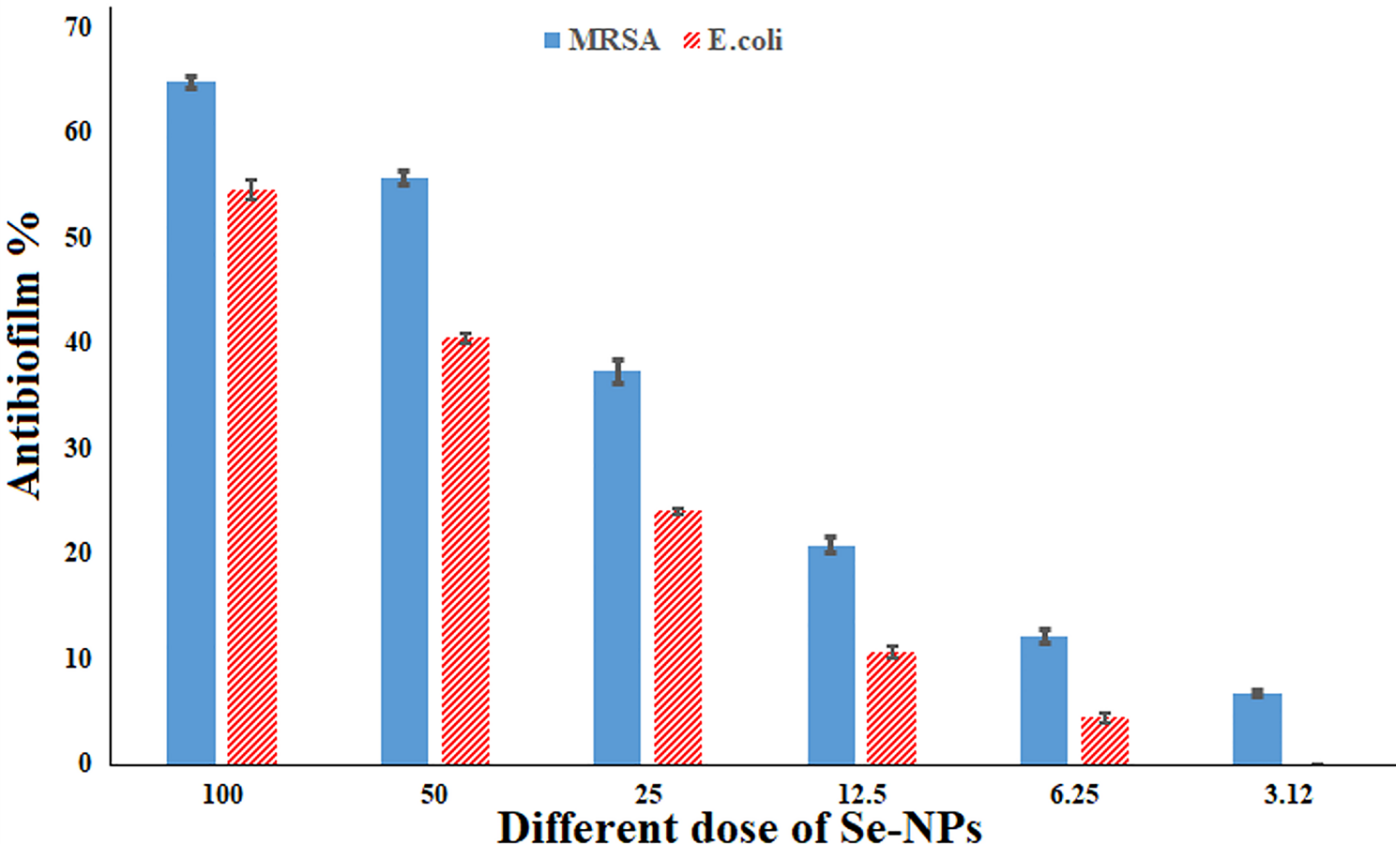
Antibiofilm assay of SeNPs versus *E.coli* and *S. aureus*

**Fig. 9:**
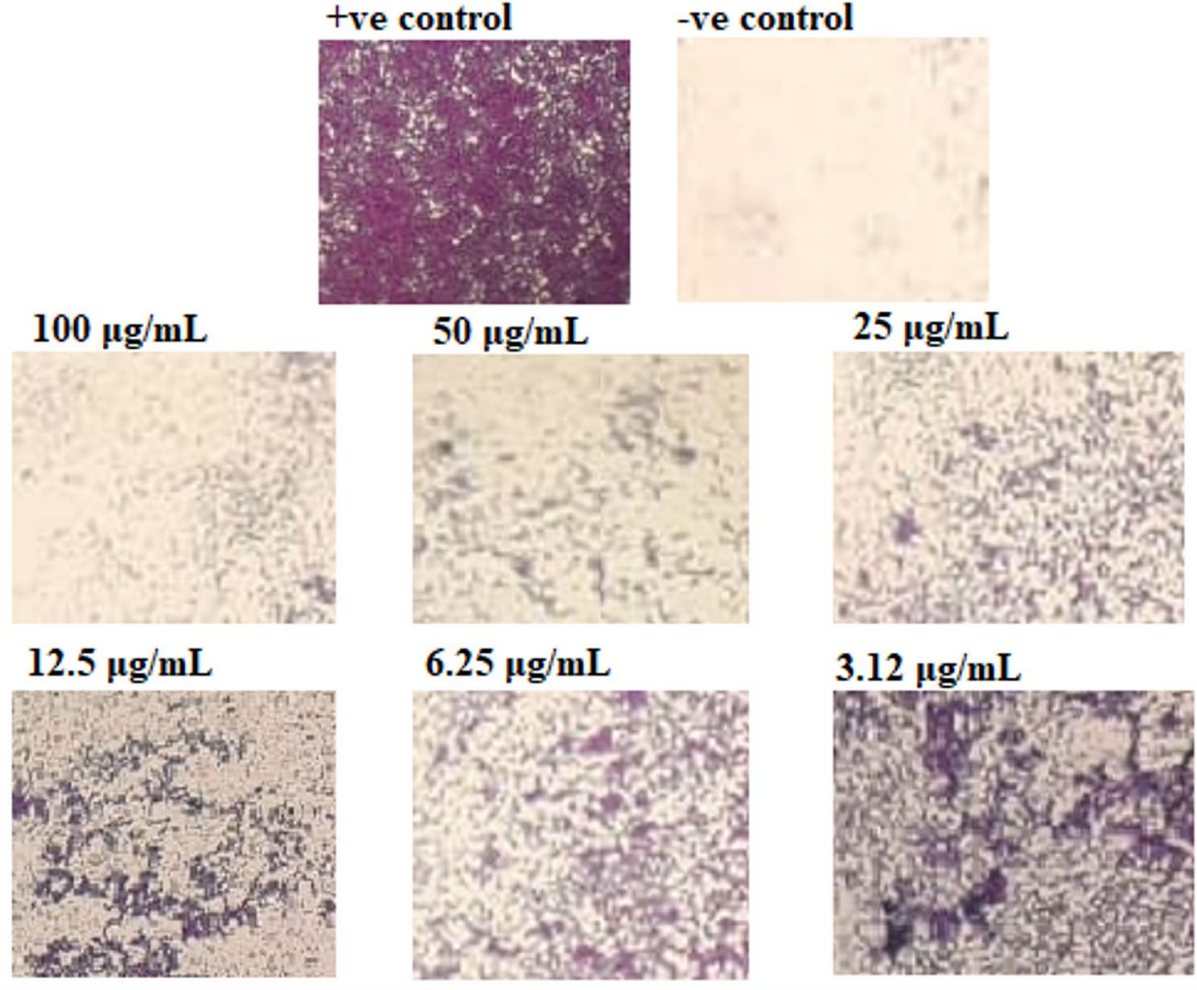
Pictures of *S. aureus* biofilm via light-inverted microscopy under different SeNPs concentrations

In this work, the antibiofilm efficiency of SeNPs was ascertained against different bacteria. Consequently, SeNPs exhibited the most effectiveness against the biofilm induced by *S. aureus* whenever employed at concentrations under the MIC limit; at 100, 50, 25, 12.5, 6.25, and 3.12 µg/mL, the creation of biofilm was reduced by 64.8, 55.76, 37.36, 20.86, 12.1, and 6.8%, respectively. Furthermore, SeNPs showed strongly biofilm reduction activity against *E*. *coli* at doses below the fatal dose without affecting the growth of bacteria, with percentages of 54.53, 40.5, 24.08, 10.72, and 4.43%, accordingly (Fig. 8). The effect of nanoparticles upon the outer structure of the biofilm matrix was examined using inverted microscopy methods Fig. 9.

Jha and colleagues examined the effectiveness of RMLP-SeNPs at concentrations ranging from 10 to 100 µg/mL in preventing the formation of biofilms by various bacteria. The RMLP-SeNPs showed an antibiofilm impact at the lowest dose of 10 µg/mL, lowering the production of biofilm for every tested microbes between 2.87 ± 1.24% to 13.14 ± 1.47% [106]. One research found that SeNPs having size ranges around 80 and 220 nm suppressed the biofilm development by 42%, 34.3%, and 53.4% when administered to *S. aureus*, *P. aeruginosa*, and *P. mirabilis*, respectively [112]. SeNPs have been proven to be highly effective antibiofilm ingredients against strains of bacteria, comprising *E. coli*,* E. faecalis*, S. *typhimurium*,* S. aureus*, and *S. enteritidis*. It was generated from *B. licheniformis* TUB5 and had sizes that ranged from 10 to 50 nm [113]. SeNPs have been proven to be highly effective antibiofilm ingredients against strains of bacteria, comprising *E. coli* [114]. Additionally, biogenic SeNPs synthesized from *B. licheniformis* JS2 successfully prevented *S. aureus* colonies from sticking to various plastic surfaces and forming biofilms [114].

### LuxS, and cna virulence gene expression

**Fig. 10:**
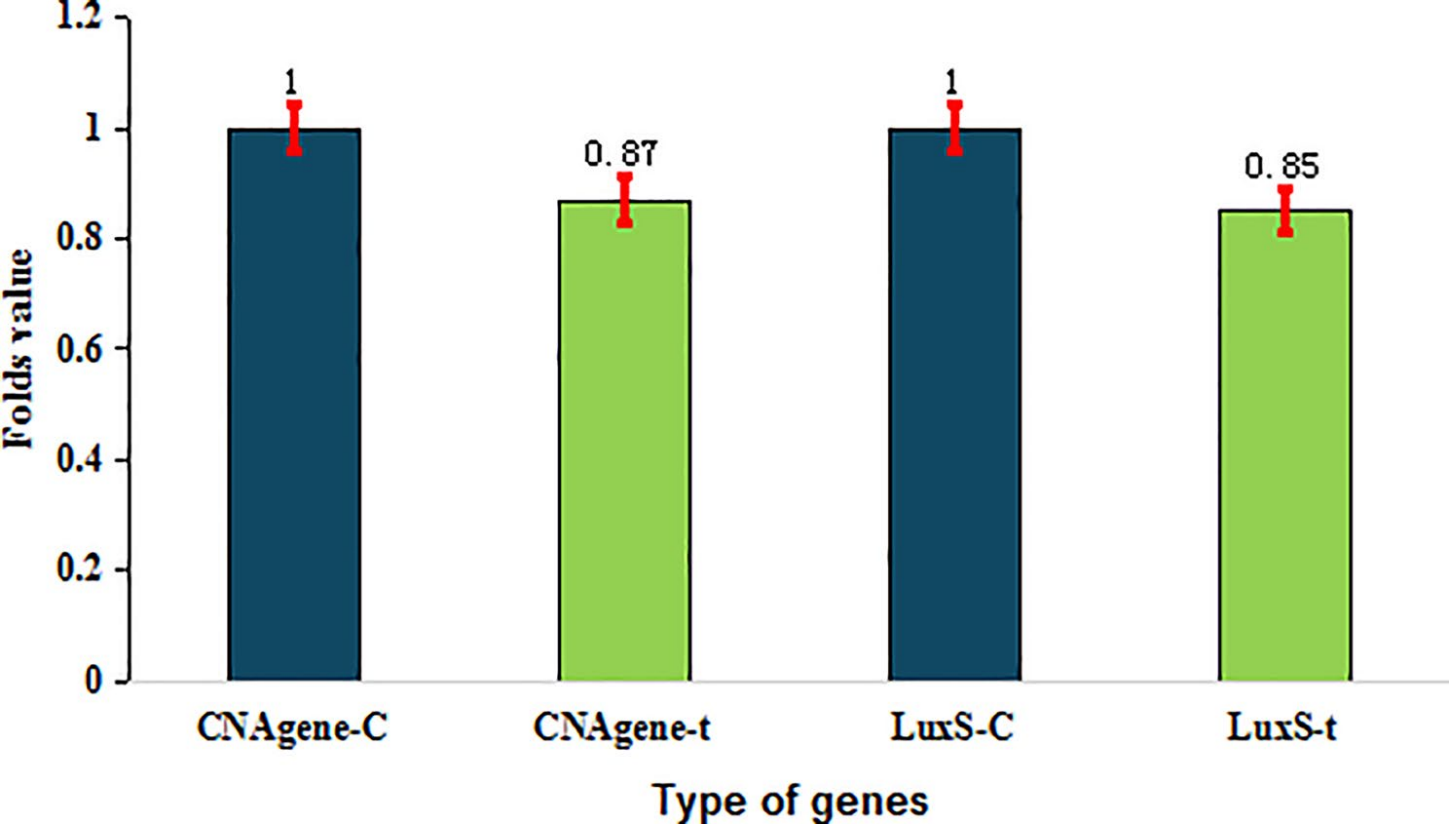
CNA, and LuxS fold change expression in control and treated cultures of *S. aureus* and *E. coli*

SeNPs concentrations in the range of 62.5–31.25 µg/mL revealed reduced expression of two essential genes required by *S. aureus* to generate biofilms, cna (0.9-fold change), and quorum sensing gene LuxS of *E. coli* (4.2 folds of control to 3.4 folds of treated) when compared with the RecA gene. The present study of SeNPs as antibacterial agent showed antibiofilm activity against *S*. *aureus* biofilm gene cna as it led to decreased expression from one fold (in control) to 0.87 folds (in treated culture) for 24 h in mannitol salt broth at 37^°^C. In case of *E. coli*, SeNPs downgraded the expression of LuxS gene responsible for quorum sensing communication between cells, decreasing from one-fold (in control) to 0.85 folds (in treated *E. coli* culture) in nutrient broth at 37^°^C for 24 h of incubation Fig. 10.

It has been determined that there are a number of protein components on microbial surfaces which are ability to communicate with the matrix of extracellular proteins of the host and detect sticky matrix components [115, 116]. Microbial surfaces generate a number of proteins, including collagen-binding (cna), fibrinogen-binding (fib), elastin-binding (ebpS), fibronectin-binding proteins A and B (fnbA and fnbB), as well as clumping factors A and B (clfA and clfB) and laminin-binding (eno) [117]. *Staphylococcus aureus* biofilm producers exhibit significant pathogenicity due of the protective advantages biofilms confer against host defenses and antibacterial agents [118]. The capacity of *S. aureus* to generate biofilms is a crucial determinant of its virulence and persistent bacterial infections [119]. Using the fnbA and cna genes, genes for biofilm formation within *S. aureus* were downregulated by plant-synthesized AgNPs [120]. Gene expression reported that, in comparison to the housekeeping gene RecA, the expression of fnbA was reduced by six times, whilst that of Cna was reduced by 12.5 times [120]. Another potential use of NPs was in green manufactured artemisinin nano-copper (ANC) therapy, which proved able to kill biofilms of bacterial species and decrease genes linked to virulence as well as biofilm in *S*. *aureus* (cna, PVL, ClfA, and femB) and *E*. *coli* (HlyA, gyrA, and F17). Green-synthesized ANC has good antibiofilm qualities and is anticipated to have both antibacterial as well as antibiofilm qualities [121]. When compared to disinfectants alone, total elimination of *S. aureus* biofilm was seen following therapy with disinfectants carried onto copper (CuNPs) and silver (AgNPs) nanoparticles at varied doses and exposure durations [122]. Biologically produced selenium nanoparticles have anti-QS and anti-biofilm characteristics against a variety of diseases [123].

### Antioxidant assessment

**Fig. 11:**
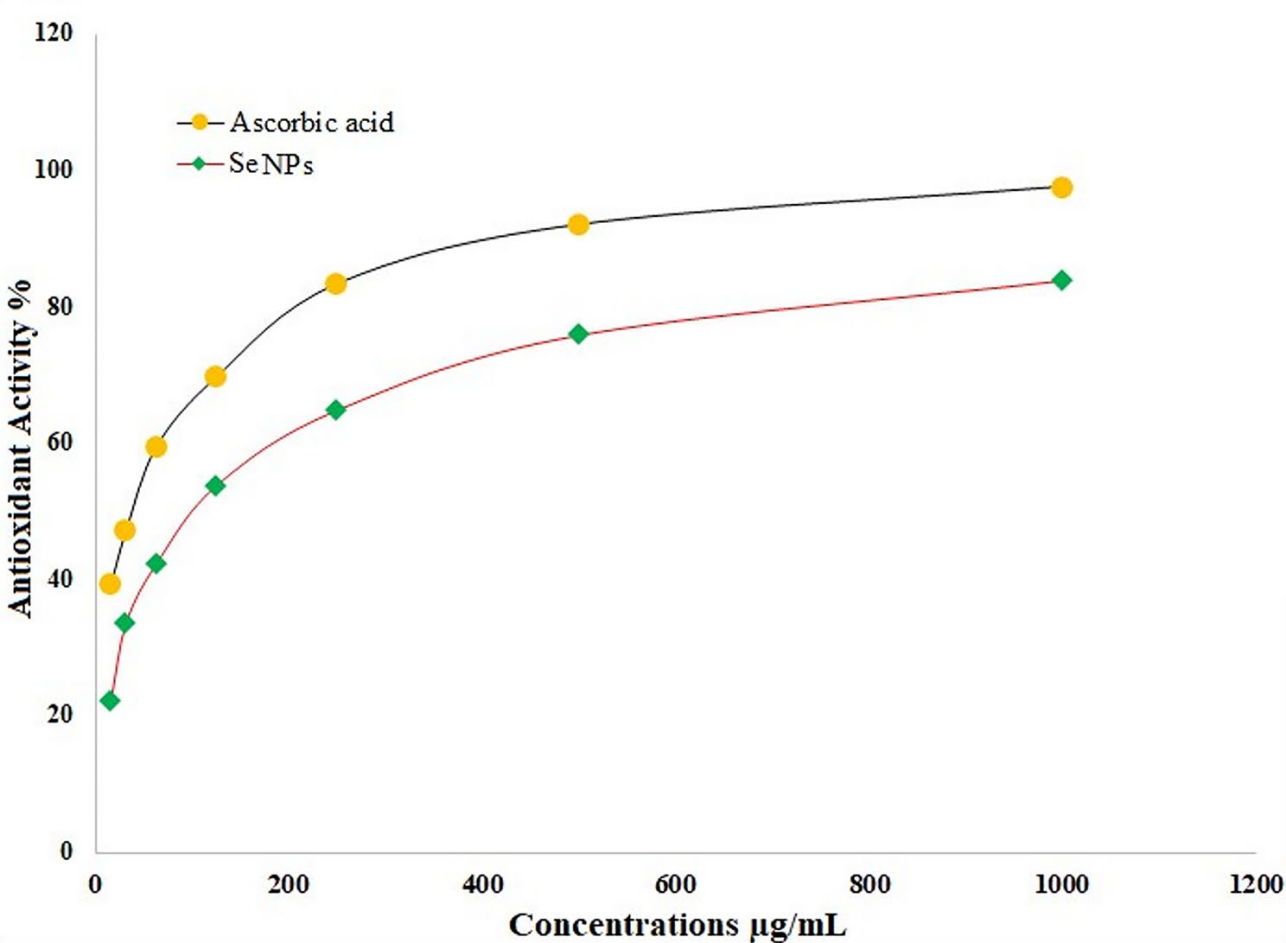
Antioxidant activity of SeNPs biofabricated via pineapple peel extract

Numerous biological activities generate reactive oxygen species (ROS), which can through oxidation destroy elements in cells and frequently result in cell death. Antioxidant substances are often used to lessen the negative effects of ROS [124]. Consequently, researchers employed antioxidant materials to lessen the negative effects of ROS. Antioxidants are also used as therapies when they exhibit antibacterial, antitumor, anti-inflammatory, and antitumor effects [79]. Figure 11 shows the antioxidant capacity of SeNPs at various doses. This study evaluated the antioxidant capabilities of SeNPs and ascorbic acid employed as a reference for comparison. The biosynthesized SeNPs exhibited significant antioxidant activity, shown by an IC_50_ level of 98.3 µg/mL, much greater than the IC_50_ level of 35.2 µg/mL for ascorbic acid. Additional researches assessed the antioxidant activity of SeNPs produced using various methods, and these investigations demonstrated the strong antioxidant effect of SeNPs [125]. In a research, the antioxidant capacity of generated SeNPs using bee propolis extracted with ethanol was evaluated, and IC_50_ was determined to be between 78.9 and 358.2 µg/mL [126]. The synthesized SeNPs’ IC_50_ was determined to be 41.5 µg/mL in another investigation [127]. By activating the enzymes SOD, CAT, GST, and PO, SeNPs may improve the antioxidative capability of aquatic animals [128]. Additionally, SeNPs reduce the amount of thiobarbituric acid reactive compounds (TBARS) in the blood, indicating less damage to fats and lower levels of MDA [128]. Consequently, phyto-synthesized SeNPs present an opportunity to function as a natural antioxidant additive in food packaging materials, potentially substituting artificial antioxidants due to their superior biocompatibility [129]. Furthermore, the SeNPs exhibit considerable capacity for neutralizing harmful free radicals, indicating substantial antioxidative opportunities [130]. It was proved that antioxidant activity utilizing the DPPH test, revealing that the IC_50_ of *E. retusa* extract was 0.054 mg/ml, whereas that of SeNPs was 0.247 mg/ml. The plant extract had far more effectiveness than the metal nanoparticle solutions, which had much lower potency [131, 132]. A comparison with previous research on a number of biological uses for SeNPs prepared from plant extracts is shown in Table 5.

**Table 5:** Compartive biological potentials of plant-based SeNPs

Plants	Biological activity	Ref.
*Rosa roxburghii*	Antioxidant, Cytotoxicity	[133]
*Ceropegia bulbulbosa* Rob	Antimicrobial	[134]
*Spirulina plantesis*	Antimicrobial, Antioxidant	[135]
*Diospyros montana*	Antimicrobial, Antioxidant	[136]
*Zingiber officinale*	Antimicrobial, Antioxidant	[99]
Hawthorn fruit	Antitumor	[137]
*Aloe vera*	Antibacterial, Antifungal	[138]
*Psidium guajava*	Antibacterial	[139]
*Withania somnifera*	Antibacterial, Antioxidant, Anticancer	[140]
Broccoli	Antioxidant, Anticancer	[141]
*Emblica officinali*	Antimicrobial	[79]
Fenugreek	Anticancer	[142]
*Azardirachta indica*	Antibacterial	[21]
*Hibiscus sabdariffa*	Antioxidant	[143]
*Leucas lavandulifolia*	Antibacterial	[144]

### Cytotoxicity and cancer prevention potential of SeNPs 

**Fig. 12:**
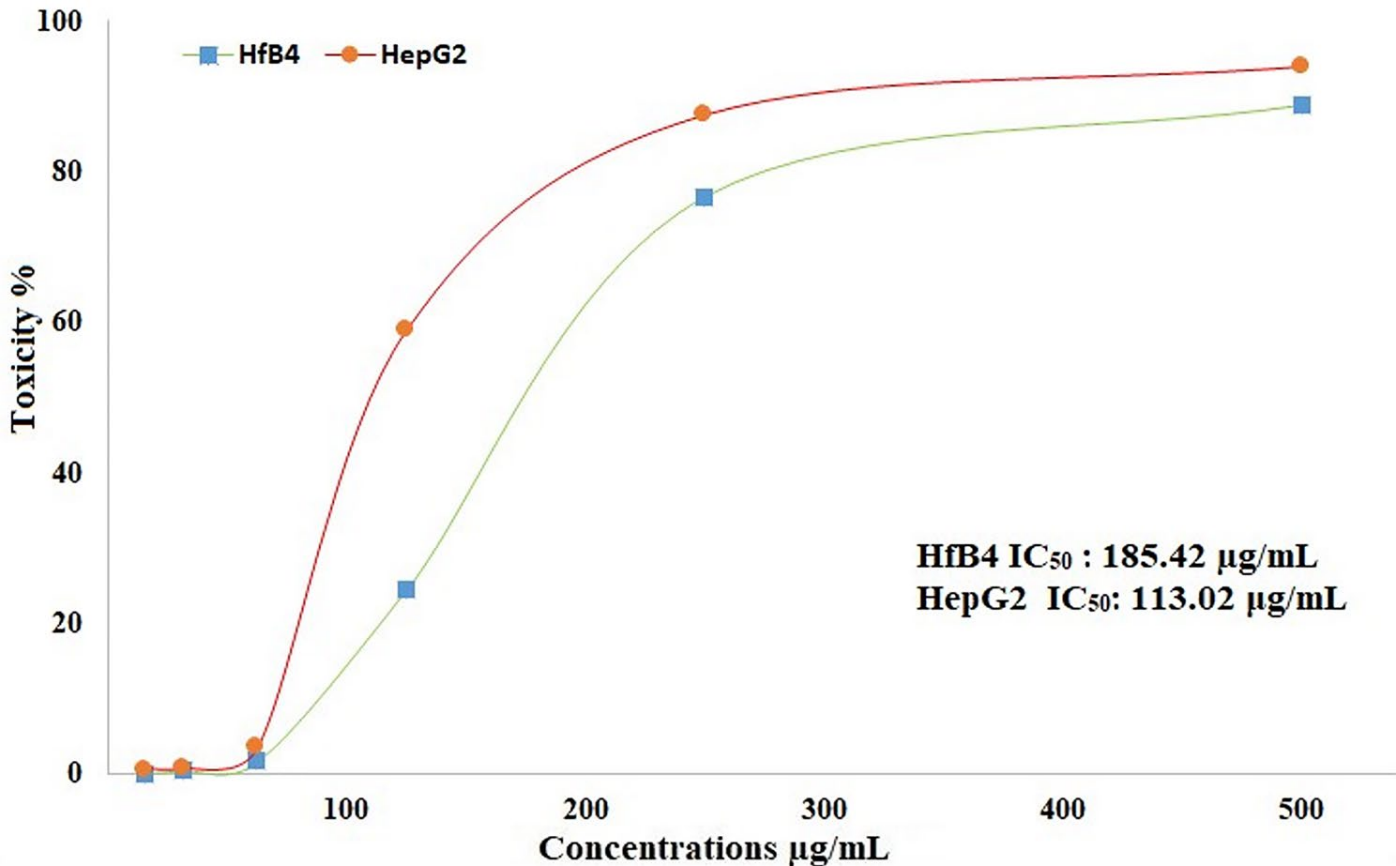
Toxicity and antitumoir activity of SeNPs generated via pineapple peel extract

Any product’s safety must be assessed by cytotoxicity upon human normal cell lines [77]. Figure 12 illustrates the results of testing the cytotoxicity of produced SeNPs against HFB4 cell line (normal foreskin). The results showed that the generated SeNPs exhibited an IC_50_ of 185.42 µg/mL against HFB4 cell line. As shown in Fig. 12, the anticancer potential of produced SeNPs was examined against the HepG2 malignant cell line at dosages that varied from 15.13 to 500 µg/mL. With an IC_50_ of 113.02 µg/mL, the produced SeNPs demonstrated promising anticancer effects based on the HepG2 malignant cell line. The process underpinning cytotoxicity, which involves the diffusion of SeNPs across the cell membrane via ion channels and their attachment to proteins inside the cell or nitrogen bases within DNA, may result in a stopped cell cycle, mitochondrial dysfunction, DNA breakage, and death [145, 146]. It has been reported that stinging nettle leaves extract-derived SeNPs possess anticancer properties toward the HepG2 cancer cell line, with an IC_50_ of 102.8 µg/mL [108]. A research demonstrated the anticancer efficacy of SeNPs derived from *Moringa oleifera* extract against Caco2, HepG2, and MCF-7, with IC_50_ values of 150.87, 392.57, and 252.44 µg/mL, respectively [103].

## Conclusion

This work utilized pineapple peel waste (PPW) towards the creation of selenium nanoparticles (SeNPs) using a novel sustainable and eco-friendly technique. Our investigation revealed that the PPW extract was a substantial source of many significant phytochemicals, particularly phenolic acids, flavonoids, tannins, flavonols, and saponins. Characterization studies indicated that the generated SeNPs were extremely crystalline, sphere shape, polydisperse, as well as ranged in size from 33 to 73 nm. Furthermore, SeNPs demonstrated potential antibacterial efficacy against hazardous bacteria, both Gram-positive and -negative. Similarly, the generated SeNPs exhibited significant antibiofilm action. SeNPs decreased the expression of two critical genes necessary for *S. aureus* biofilm formation, cna (0.9 fold change) along with the quorum sensing gene LuxS of *E. coli* (4.2 folds in control vs. 3.4 folds in treatment), relative to the RecA gene. Moreover, the SeNPs demonstrated anticancer efficacy against the HepG2 malignant cell line. Eventually, synthesised SeNPs will have significant antibacterial, antibiofilm, antioxidant, and anticancer properties, which will make possible their use in the medical field. Although nanoparticles have the potential to revolutionise a number of industries, obstacles to their widespread use must be overcome creatively. Scalability are major obstacles, especially when transferring from lab to manufacturing facilities.

### Limitations

Challenges in Targeted Delivery: Precise delivery to specific tissues is difficult, leading to potential side effects and systemic toxicity.

Toxicity Despite Low Harm Profile: Although SeNPs are generally less toxic than other nanoparticles, some studies report significant in vitro and in vivo toxicity.

Inconsistent Synthesis Processes: Industrial-scale production suffers from inconsistent methods and poor batch-to-batch reproducibility.

Polydispersity and Scalability Issues: Nanoparticles often vary in size and shape, making large-scale, uniform production challenging.

Limited Long-Term Stability Data: The long-term safety, stability, and behavior of SeNPs in the body remain under-researched.

Lack of Standard Animal Models: Current models are inadequate for evaluating therapeutic effects and immune system interactions, especially immunotoxicity.

Unsuitable Screening Protocols: Standard drug testing methods don’t fit nanomedicine; each formulation needs a custom evaluation approach.

Regulatory Gaps: There are no well-defined regulatory guidelines for the characterization and clinical approval of SeNPs.

Underdeveloped Industrial Infrastructure: The transition from lab-scale research to industrial production is limited by lack of equipment and quality control systems.

Difficulty in Clinical Translation: Translating promising lab results into clinical success is still hindered by multiple unresolved scientific and regulatory barriers.

### Alignment with sustainable development goals (SDGs)

SDG 3 – Good Health and Well-being.

This study supports efforts to improve public health by exploring the antibacterial properties of green-synthesized selenium nanoparticles (SeNPs), contributing to the development of safer and more effective therapeutic solutions.

SDG 9 – Industry, Innovation, and Infrastructure.

The use of natural plant extracts in the formulation of SeNPs promotes sustainable innovation and advances eco-friendly nanotechnology.

SDG 15 – Life on Land.

Green synthesis methods help protect ecosystems and biodiversity by reducing the environmental impact associated with conventional nanoparticle production.

SDG 17 – Partnerships for the Goals.

The research encourages interdisciplinary collaboration, fostering innovation and shared knowledge in support of global sustainability goals.

### Futuristic studies

Toxicity Evaluation: Further studies are needed to understand and minimize the nanotoxicity of SeNPs to ensure safe biomedical use.

Specific Antibacterial Action: Research should focus on identifying the precise antibacterial mechanisms of SeNPs, alone or combined with other therapies.

Mechanistic Studies: Investigate the molecular pathways through which SeNPs exert antibacterial and therapeutic effects.

In Vivo Testing and Biocompatibility: More animal studies are required to evaluate SeNPs’ safety, side effects, and compatibility with biological systems.

Advancing Theranostics: Develop SeNP-based theranostic systems for combined diagnosis and therapy, a field still in early stages.

Personalized Medicine Potential: Utilize SeNPs for tailored treatments, leveraging their antioxidant and anti-inflammatory properties to match individual patient profiles.

AI and Machine Learning Integration: Apply AI/ML to optimize SeNP synthesis, predict ideal experimental conditions, and design nanoparticles with targeted properties.

Standardization and Regulatory Frameworks: Establish clear guidelines for production, safety, and pharmacological profiling to support clinical translation.

Sustainable and Scalable Production: Develop eco-friendly, high-yield synthesis methods that ensure consistency and industrial scalability.

Diagnostics and Biomarker Identification: Use SeNPs in diagnostic tools for tracking treatment efficacy and identifying disease-related biomarkers.

Commercial and Industrial Applications: Explore SeNP use in clinical, environmental, and industrial settings, with potential for commercialization and patenting.

## Data Availability

The data and materials of this study have been presented in the manuscript.
